# Supplementary figures and images for: Longitudinal study of Chlamydia pecorum in a healthy Swiss cattle population
Source: PLoS One. 2023 Dec 11;18(12):e0292509. doi: 10.1371/journal.pone.0292509 (PMC10712897; doi:10.1371/journal.pone.0292509)

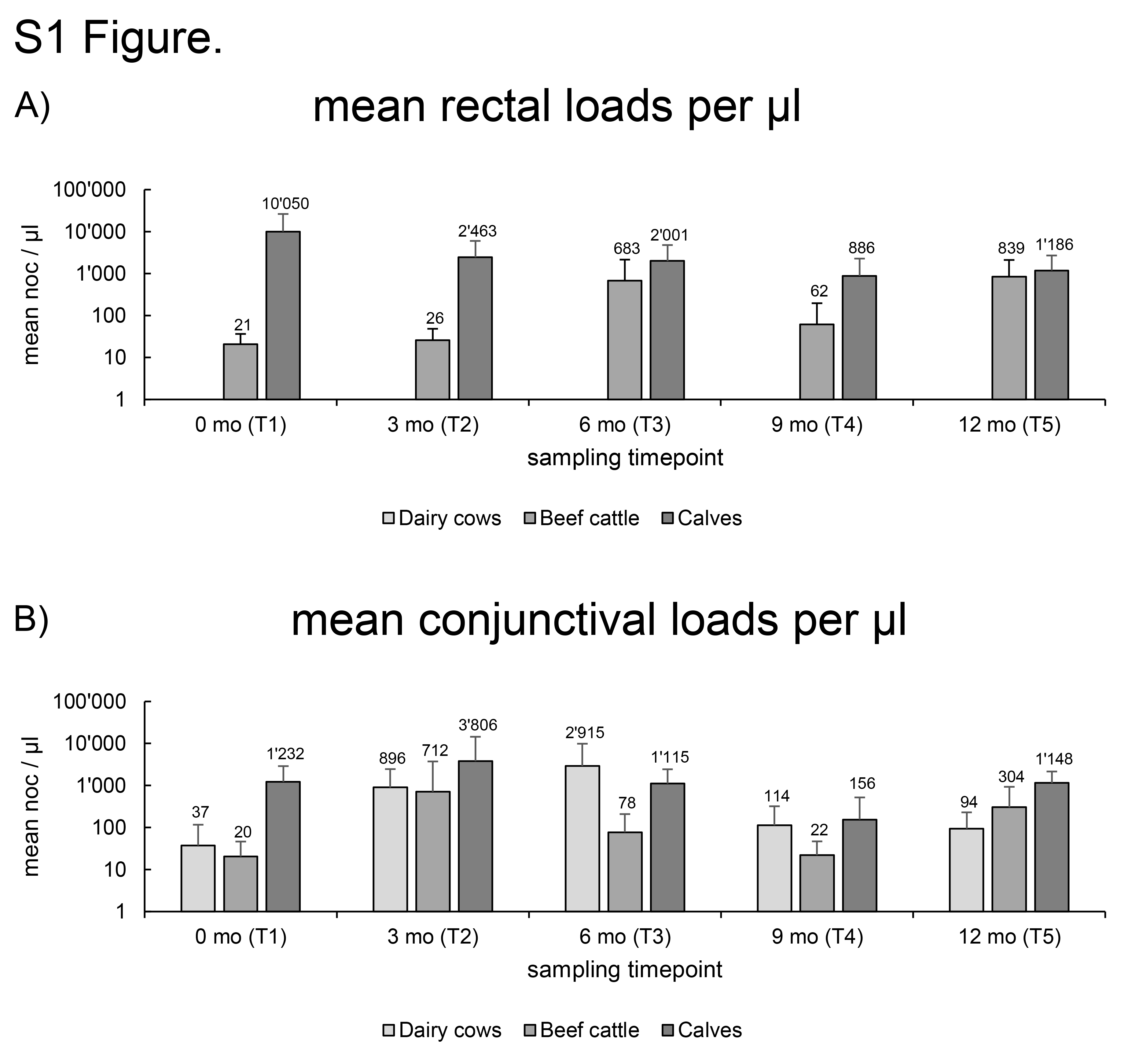

Supplement: S1 Fig — Shown are the mean (A) rectal and (B) conjunctival C. pecorum loads as number of copies (noc) per μl for each age category at all five sampling timepoint (logarithmic scale, mean ± standard deviation). (TIF) [file pone.0292509.s001.tif]

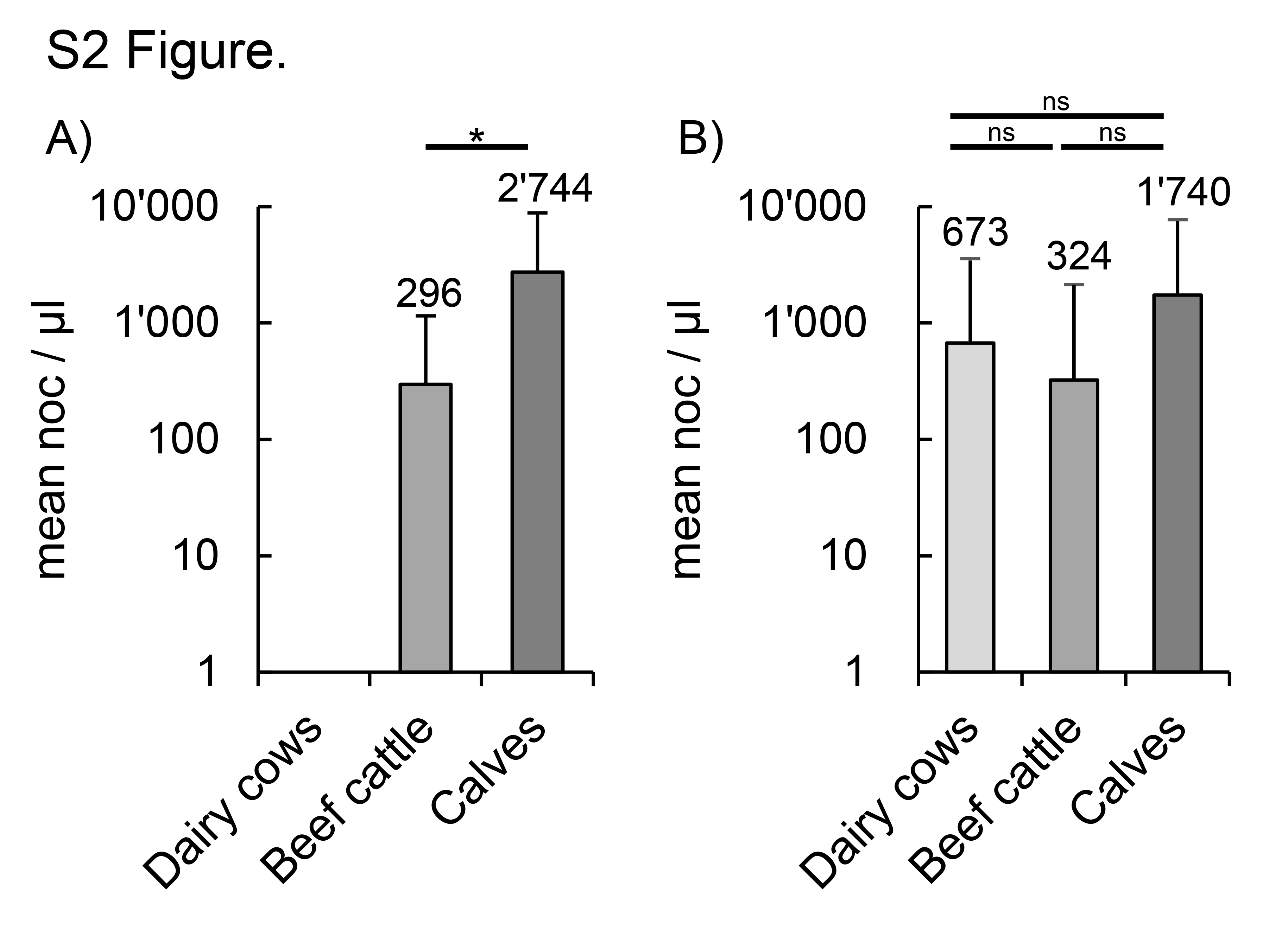

Supplement: S2 Fig — Shown is the (A) rectal and (B) conjunctival C. pecorum load as number of copies (noc) per μl for each age category including all sampling timepoints (logarithmic scale, mean ± standard deviation). Statistical analyses were performed using the absolute loads of each respective sampling timepoint. Significant differences were represented with asterisks: Three asterisks (***) represent p-values <0.001, two asterisks (**) represent p-values <0.01 and one (*) represents p-values between 0.01 and 0.05. Non-significant values were labeled with ns. (TIF) [file pone.0292509.s002.tif]

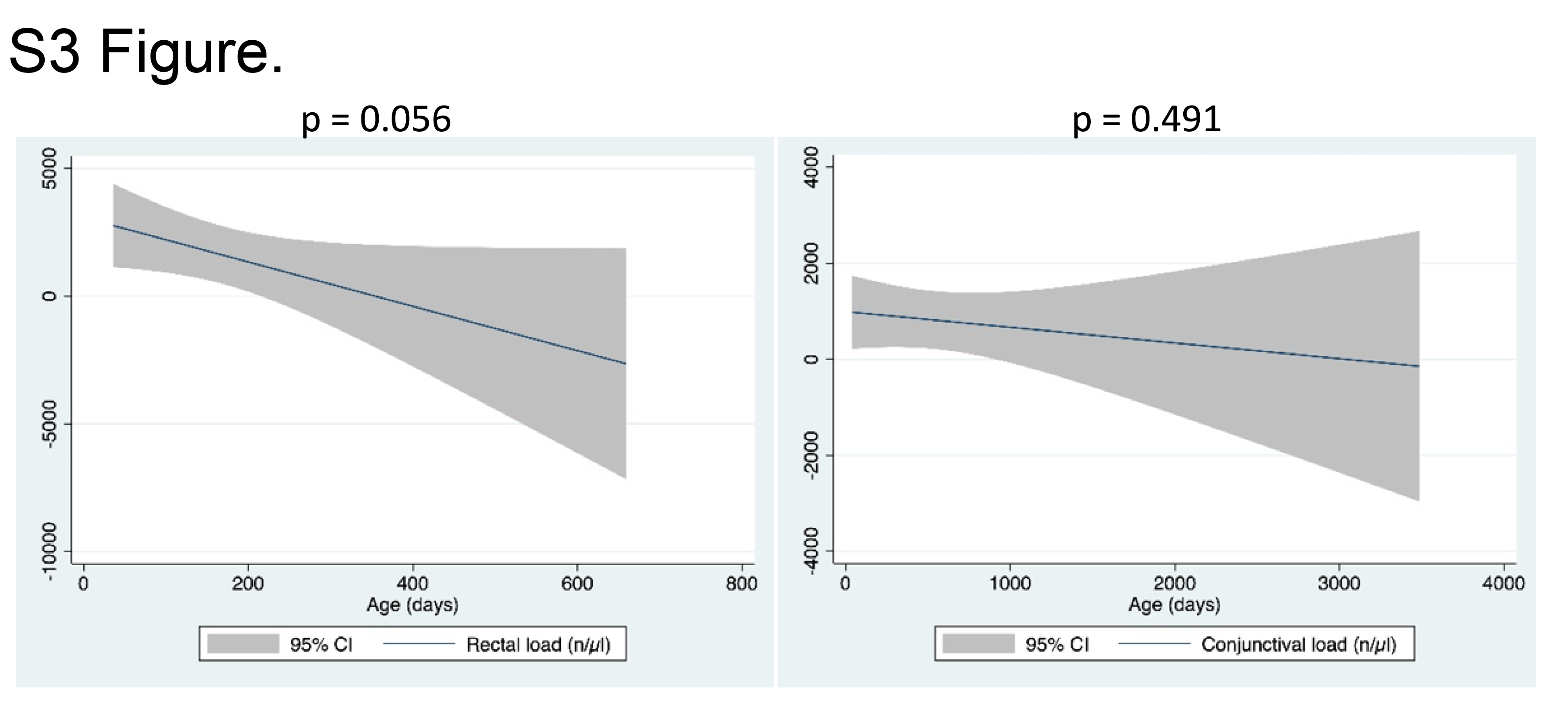

Supplement: S3 Fig — In this figure, the correlation between age and absolute loads are shown at the rectal (left panel) and at the conjunctival site (right panel). A regression analysis using a confidence interval of 95% was performed. (TIF) [file pone.0292509.s003.tif]
