# Supplementary material for: Longitudinal study of Chlamydia pecorum in a healthy Swiss cattle population
Source: PLoS One. 2023 Dec 11;18(12):e0292509. doi: 10.1371/journal.pone.0292509 (PMC10712897; doi:10.1371/journal.pone.0292509)
Supplement: S1 Table — A total of 308 bovines were sampled at least one time. The dairy herd was the most consistent age category with 34 animals tested at all five sampling timepoints. (DOCX) [file pone.0292509.s004.docx]

| N of samplings | Dairy cows | Beef cattle | Calves | Total |
| --- | --- | --- | --- | --- |
| 1 | 26 | 45 | 35 | 106 |
| 2 | 18 | 30 | 8 | 56 |
| 3 | 34 | 16 | 5 | 55 |
| 4 | 47 | 5 | 4 | 56 |
| 5 | 34 | 1 | 0 | 35 |
