# Supplementary material for: Longitudinal study of Chlamydia pecorum in a healthy Swiss cattle population
Source: PLoS One. 2023 Dec 11;18(12):e0292509. doi: 10.1371/journal.pone.0292509 (PMC10712897; doi:10.1371/journal.pone.0292509)
Supplement: S2 Table — Overview of the sample sizes for each age category at all five sampling timepoints. (DOCX) [file pone.0292509.s005.docx]

|  | 09.04.2021 | 05.07.2021 | 27.09.2021 | 20.12.2021 | 17.05.2022 |
| --- | --- | --- | --- | --- | --- |
| Dairy cows | 123 | 99 | 135 | 108 | 88 |
| Beef cattle | 46 | 39 | 42 | 17 | 25 |
| Calves | 5 | 15 | 12 | 20 | 8 |
| Total | 174 | 153 | 189 | 145 | 121 |
