# Supplementary material for: Longitudinal study of Chlamydia pecorum in a healthy Swiss cattle population
Source: PLoS One. 2023 Dec 11;18(12):e0292509. doi: 10.1371/journal.pone.0292509 (PMC10712897; doi:10.1371/journal.pone.0292509)
Supplement: S3 Table — Summary of primer and probe used for qPCR (Chlamydiaceae and C. pecorum) as well as for the internal amplification control. (DOCX) [file pone.0292509.s006.docx]

| Primer and Probe used for 23S *Chlamydiaceae* real-time qPCR | | |
| --- | --- | --- |
| Forward primer | Ch23S-F | 5’-CTGAAACCAGTAGCTTATAAGCGGT-3’ |
| Reverse primer | Ch23S-R | 5’-ACCTCGCCGTTTAACTTAACTCC-3’ |
| Probe | Ch23S-p | FAM-CTCATCATGCAAAAGGCACGCCG-TAMRA |
| Primer and Probe used for *C. pecorum-specific* real-time qPCR | | |
| Forward primer | CppecOMP1-F | 5’-CCATGTGATCCTTGCGCTACT-3’ |
| Reverse primer | CppecOMP1-R | 5’-TGTCGAAAACATAATCTCCGTAAAAT-3’ |
| Probe | CppecOMP1-S | FAM-TGCGACGCGATTAGCTTACGCGTAG-TAMRA |
| Primer and Probe used for internal control (eGFP) | | |
| Forward primer | EGFP-1-F | 5’-GACCACTACCAGCAGAACAC-3’ |
| Reverse primer | EGFP-10-R | 5’-CTTGTACAGCTCGTCCATGC-3’ |
| Probe | EGFP-HEX | HEX-AGCACCCAGTCCGCCCTGAGCA-BHQ1 |
