# Supplementary material for: Longitudinal study of Chlamydia pecorum in a healthy Swiss cattle population
Source: PLoS One. 2023 Dec 11;18(12):e0292509. doi: 10.1371/journal.pone.0292509 (PMC10712897; doi:10.1371/journal.pone.0292509)
Supplement: S4 Table — LOD performed with the wildtype C. pecorum strain “W73” and with the C. pecorum standard used in this study. (DOCX) [file pone.0292509.s007.docx]

| W73 | Ct1 | Ct2 | Ct3 | Ct4 | Ct5 | Ct6 | Ct7 | Ct8 | Ct9 | Ct10 | Rate | Ctmean | SD |
| --- | --- | --- | --- | --- | --- | --- | --- | --- | --- | --- | --- | --- | --- |
| 50 | 32,67 | 32,69 | 31,92 | 33,00 | 32,20 | 33,18 | 32,08 | 33,62 | 32,50 | 32,68 | 100% | 32,65 | 0,52 |
| 20 | 34,47 | 33,76 | 33,42 | 33,71 | 34,12 | 34,32 | 34,35 | 35,50 | 34,07 | 34,41 | 100% | 34,21 | 0,57 |
| 10 | 34,61 | 35,00 | 35,50 | 35,12 | 35,93 | 34,76 | 35,46 | 34,93 | 35,31 | 35,46 | 100% | 35,21 | 0,40 |
| 5 | 35,48 | 35,77 | 37,42 | 36,67 | 35,15 | 36,66 | 35,85 | 36,18 | 36,01 | 36,53 | 100% | 36,17 | 0,67 |
| 2 | 37,36 | 36,05 | 36,93 | 36,73 | 37,85 | 39,32 | 39,18 | 36,69 | 36,61 |  | 90% | 37,41 | 1,15 |
| 1 | 39,67 | 38,59 | 35,24 | 37,61 | 37,33 | 38,23 | 36,10 |  |  |  | 70% | 37,54 | 1,50 |
| Cpec | Ct1 | Ct2 | Ct3 | Ct4 | Ct5 | Ct6 | Ct7 | Ct8 | Ct9 | Ct10 | Rate | Ctmean | SD |
| 50 | 32,56 | 32,73 | 32,61 | 32,58 | 32,86 | 32,79 | 32,68 | 33,35 | 32,60 | 32,12 | 100% | 32,69 | 0,31 |
| 20 | 33,29 | 33,39 | 34,09 | 33,27 | 33,74 | 34,69 | 34,04 | 34,32 | 34,63 | 33,18 | 100% | 33,86 | 0,57 |
| 10 | 34,89 | 34,97 | 34,56 | 35,34 | 34,85 | 34,66 | 35,41 | 35,35 | 35,41 | 34,45 | 100% | 34,99 | 0,37 |
| 5 | 36,51 | 37,03 | 35,28 | 35,60 | 36,77 | 36,97 | 35,78 | 35,15 | 36,12 | 35,33 | 100% | 36,05 | 0,73 |
| 2 | 38,66 | 37,52 | 37,12 | 37,93 | 37,27 | 37,41 | 35,82 | 37,17 | 37,21 | 36,38 | 100% | 37,25 | 0,77 |
| 1 | 38,48 | 39,11 | 38,50 | 36,66 |  |  |  |  |  |  | 40% | 38,19 | 1,06 |
