# Supplementary material for: Longitudinal study of Chlamydia pecorum in a healthy Swiss cattle population
Source: PLoS One. 2023 Dec 11;18(12):e0292509. doi: 10.1371/journal.pone.0292509 (PMC10712897; doi:10.1371/journal.pone.0292509)
Supplement: S5 Table — P-values for comparison of different prevalences between age categories are shown. The age categories are abbreviated as d (dairy cows), b (beef cattle) and c (calves). Comparisons were considered significant if the p-value was < 0.05. (DOCX) [file pone.0292509.s008.docx]

|  | Animal prevalence | | | Rectal prevalence | | | Conjunctival prevalence | | |
| --- | --- | --- | --- | --- | --- | --- | --- | --- | --- |
| Timepoint | b>d | c>d | c>b | b>d | c>d | c>b | b>d | c>d | c>b |
| T1 | <0.001 | <0.001 | 0.014 | <0.001 | <0.001 | 0.004 | <0.001 | <0.001 | 0.007 |
| T2 | <0.001 | <0.001 | 0.005 | 0.001 | <0.001 | <0.001 | <0.001 | <0.001 | 0.062 |
| T3 | <0.001 | <0.001 | 0.008 | <0.001 | <0.001 | 0.002 | <0.001 | <0.001 | 0.165 |
| T4 | <0.001 | <0.001 | 1 | <0.001 | <0.001 | 0.161 | 0.059 | <0.001 | 0.173 |
| T5 | <0.001 | <0.001 | 0.031 | 0.002 | <0.001 | 0.001 | <0.001 | <0.001 | 0.012 |
