# Supplementary material for: Longitudinal study of Chlamydia pecorum in a healthy Swiss cattle population
Source: PLoS One. 2023 Dec 11;18(12):e0292509. doi: 10.1371/journal.pone.0292509 (PMC10712897; doi:10.1371/journal.pone.0292509)
Supplement: S6 Table — P-values for comparisons between age and positivity for animal and sample prevalence are shown. For these calculations all age categories were included evaluated at all five sampling timepoints. Comparisons were considered significant if the p-value was < 0.05. (DOCX) [file pone.0292509.s009.docx]

| Timepoint | Animal | Rectal | Conjunctival |
| --- | --- | --- | --- |
| T1 | <0.0001 | 0.0001 | <0.0001 |
| T2 | <0.0001 | <0.0001 | <0.0001 |
| T3 | <0.0001 | <0.0001 | <0.0001 |
| T4 | <0.0001 | <0.0001 | 0.0002 |
| T5 | <0.0001 | <0.0001 | <0.0001 |
