# Supplementary material for: Longitudinal study of Chlamydia pecorum in a healthy Swiss cattle population
Source: PLoS One. 2023 Dec 11;18(12):e0292509. doi: 10.1371/journal.pone.0292509 (PMC10712897; doi:10.1371/journal.pone.0292509)
Supplement: S7 Table — P-values for comparisons of animal prevalences with age within each age category and sampling timepoint. Comparisons were considered significant if the p-value was < 0.05. (DOCX) [file pone.0292509.s010.docx]

| Timepoint | Dairy cows | Beef cattle | Calves |
| --- | --- | --- | --- |
| T1 | 0.2543 | 0.0121 | na |
| T2 | 0.1234 | 0.8777 | na |
| T3 | 0.0677 | <0.001 | 0.0524 |
| T4 | 0.5182 | 0.9094 | 0.7476 |
| T5 | 0.1638 | 0.1446 | na |
