# Supplementary material for: Longitudinal study of Chlamydia pecorum in a healthy Swiss cattle population
Source: PLoS One. 2023 Dec 11;18(12):e0292509. doi: 10.1371/journal.pone.0292509 (PMC10712897; doi:10.1371/journal.pone.0292509)
Supplement: S8 Table — P-values for comparisons between C. pecorum positivity rates (animal prevalence) with breed, lactation status and lactation number at each sampling timepoint. Comparison with breed was performed in each age category whereas the other comparisons were only performed in dairy cows. Comparisons were considered significant if the p-value was < 0.05. (DOCX) [file pone.0292509.s011.docx]

| Timepoint | Breed | | | Lactation vs. galt | Lactation number |
| --- | --- | --- | --- | --- | --- |
|  | Dairy cows | Beef cattle | Calves | Only dairy cows | Only dairy cows |
| T1 | 0.349 | 0.590 | na | 1 | 0.822 |
| T2 | 0.527 | 0.512 | na | 1 | 0.636 |
| T3 | 0.476 | 0.132 | 0.818 | 0.589 | 0.469 |
| T4 | 0.805 | 0.579 | 0.453 | 1 | 0.207 |
| T5 | 0.605 | 0.607 | na | 0.345 | 0.868 |
