# Supplementary material for: Longitudinal study of Chlamydia pecorum in a healthy Swiss cattle population
Source: PLoS One. 2023 Dec 11;18(12):e0292509. doi: 10.1371/journal.pone.0292509 (PMC10712897; doi:10.1371/journal.pone.0292509)
Supplement: S9 Table — Listed are the mean C. pecorum loads per μl with corresponding standard deviation (SD) for each age category, anatomical site and at each sampling timepoint. (DOCX) [file pone.0292509.s012.docx]

| Dairy cows | | | | |
| --- | --- | --- | --- | --- |
|  | Rectal | | Conjunctival | |
|  | Mean | SD | Mean | SD |
| T1 | - | - | 37.2 | 80.2 |
| T2 | - | - | 895.5 | 1'557.8 |
| T3 | - | - | 2'915.2 | 6'987.4 |
| T4 | - | - | 113.5 | 206.1 |
| T5 | - | - | 93.7 | 135.6 |
| Total | - | - | 672.8 | 2'902.7 |
| Beef cattle | | | | |
|  | Rectal | | Conjunctival | |
|  | Mean | SD | Mean | SD |
| T1 | 20.9 | 15.4 | 20.4 | 25.9 |
| T2 | 25.9 | 22.5 | 712.2 | 3'040 |
| T3 | 682.9 | 1'459.8 | 77.5 | 132.4 |
| T4 | 61.6 | 135.7 | 22 | 24.9 |
| T5 | 839.1 | 1'275.6 | 304.2 | 627 |
| Total | 296.2 | 856.7 | 323.6 | 1'813.6 |
| Calves | | | | |
|  | Rectal | | Conjunctival | |
|  | Mean | SD | Mean | SD |
| T1 | 10'049.7 | 16'406.6 | 1'232.3 | 1'663.4 |
| T2 | 2'462.5 | 3'584.9 | 3'806.1 | 10'644.7 |
| T3 | 2'001.2 | 2'795.9 | 1'114.6 | 1'314.7 |
| T4 | 886.3 | 1'387.2 | 155.5 | 365.1 |
| T5 | 1'185.9 | 1'514 | 1'148.2 | 1'006.5 |
| Total | 2'743.7 | 6'100.3 | 1'739.7 | 5'997.5 |
