# Supplementary material for: Longitudinal study of Chlamydia pecorum in a healthy Swiss cattle population
Source: PLoS One. 2023 Dec 11;18(12):e0292509. doi: 10.1371/journal.pone.0292509 (PMC10712897; doi:10.1371/journal.pone.0292509)
Supplement: S10 Table — P-values of the correlation between the C. pecorum load and age are shown. Absolute numbers of copies (noc) as well as mean values were used for calculations in each age category and including all bovines. Comparisons were considered significant if the p-value was < 0.05. (DOCX) [file pone.0292509.s013.docx]

| Category | Rectal | | Conjunctival | |
| --- | --- | --- | --- | --- |
|  | Absolut noc | Mean | Absolut noc | Mean |
| Dairy cows | na | na | p = 0.683 | p = 0.261 |
| Beef cattle | p = 0.232 | p = 0.141 | p = 0.995 | p = 0.789 |
| Calves | p = 0.523 | p = 0.825 | p = 0.204 | p = 0.013 |
| All categories | p = 0.056 | p < 0.001 | p = 0.491 | p = 0.028 |
