# Supplementary material for: Longitudinal study of Chlamydia pecorum in a healthy Swiss cattle population
Source: PLoS One. 2023 Dec 11;18(12):e0292509. doi: 10.1371/journal.pone.0292509 (PMC10712897; doi:10.1371/journal.pone.0292509)
Supplement: S11 Table — P-values of the comparison between the C. pecorum load (absolute numbers as well mean values) and the anatomical localization within each age category and including all bovines are shown. Comparisons were considered significant if the p-value was < 0.05. (DOCX) [file pone.0292509.s014.docx]

| Category | Absolut numbers | Mean values |
| --- | --- | --- |
| Dairy cows | na | na |
| Beef cattle | P = 0.9374 | P = 0.6953 |
| Calves | P = 0.4703 | P = 0.0429 |
| All categories | P = 0.1840 | P = 0.0009 |
