# Supplementary material for: Longitudinal study of Chlamydia pecorum in a healthy Swiss cattle population
Source: PLoS One. 2023 Dec 11;18(12):e0292509. doi: 10.1371/journal.pone.0292509 (PMC10712897; doi:10.1371/journal.pone.0292509)
Supplement: S1 Text — To test for a correlation between age and loads, a regression analysis was performed across all animals calculated with the following formula: Loads = a + bx. (DOCX) [file pone.0292509.s015.docx]

----------------------------------------------------------------------------------

----------------------------------------------------------------------------------

name: <unnamed>

log: /Users/gyni/Extern/S Löhrer/Stata/Log/230706.smcl

log type: smcl

resumed on: 6 Jul 2023, 10:10:28

. regress Amount_µl_R Age

Source | SS df MS Number of obs = 65

-------------+---------------------------------- F(1, 63) = 3.78

Model | 78375290.7 1 78375290.7 Prob > F = 0.0562

Residual | 1.3049e+09 63 20713049 R-squared = 0.0567

-------------+---------------------------------- Adj R-squared = 0.0417

Total | 1.3833e+09 64 21614021.5 Root MSE = 4551.2

------------------------------------------------------------------------------

Amount_µl_R | Coefficient Std. err. t P>|t| [95% conf. interval]

-------------+----------------------------------------------------------------

Age | -8.682465 4.463499 -1.95 0.056 -17.60206 .2371267

_cons | 3075.518 939.7494 3.27 0.002 1197.578 4953.457

------------------------------------------------------------------------------

. regress Amount_µl_A Age

Source | SS df MS Number of obs = 151

-------------+---------------------------------- F(1, 149) = 0.48

Model | 6695783.32 1 6695783.32 Prob > F = 0.4905

Residual | 2.0884e+09 149 14016348.3 R-squared = 0.0032

-------------+---------------------------------- Adj R-squared = -0.0035

Total | 2.0951e+09 150 13967544.6 Root MSE = 3743.8

------------------------------------------------------------------------------

Amount_µl_A | Coefficient Std. err. t P>|t| [95% conf. interval]

-------------+----------------------------------------------------------------

Age | -.3266939 .4726696 -0.69 0.491 -1.260695 .6073076

_cons | 993.2449 397.3762 2.50 0.014 208.0242 1778.466

------------------------------------------------------------------------------

. regress Mean_R Age

Source | SS df MS Number of obs = 65

-------------+---------------------------------- F(1, 63) = 14.11

Model | 64661573.9 1 64661573.9 Prob > F = 0.0004

Residual | 288621214 63 4581289.1 R-squared = 0.1830

-------------+---------------------------------- Adj R-squared = 0.1701

Total | 353282787 64 5520043.55 Root MSE = 2140.4

------------------------------------------------------------------------------

Mean_R | Coefficient Std. err. t P>|t| [95% conf. interval]

-------------+----------------------------------------------------------------

Age | -7.88636 2.099169 -3.76 0.000 -12.08121 -3.691506

_cons | 2941.515 441.9609 6.66 0.000 2058.326 3824.703

------------------------------------------------------------------------------

. regress Mean_A Age

Source | SS df MS Number of obs = 151

-------------+---------------------------------- F(1, 149) = 4.95

Model | 6156922.29 1 6156922.29 Prob > F = 0.0276

Residual | 185401195 149 1244303.32 R-squared = 0.0321

-------------+---------------------------------- Adj R-squared = 0.0256

Total | 191558118 150 1277054.12 Root MSE = 1115.5

------------------------------------------------------------------------------

Mean_A | Coefficient Std. err. t P>|t| [95% conf. interval]

-------------+----------------------------------------------------------------

Age | -.3132724 .1408327 -2.22 0.028 -.5915596 -.0349852

_cons | 986.0009 118.3989 8.33 0.000 752.0431 1219.959

------------------------------------------------------------------------------

. * Reverse Var, Quality control

. regress Age Mean_A

Source | SS df MS Number of obs = 151

-------------+---------------------------------- F(1, 149) = 4.95

Model | 2016427.34 1 2016427.34 Prob > F = 0.0276

Residual | 60719954.1 149 407516.47 R-squared = 0.0321

-------------+---------------------------------- Adj R-squared = 0.0256

Total | 62736381.4 150 418242.543 Root MSE = 638.37

------------------------------------------------------------------------------

Age | Coefficient Std. err. t P>|t| [95% conf. interval]

-------------+----------------------------------------------------------------

Mean_A | -.1025985 .0461235 -2.22 0.028 -.1937392 -.0114578

_cons | 623.5495 64.17551 9.72 0.000 496.7379 750.3612

------------------------------------------------------------------------------

. * resulting in same p-value

. by Category2 , sort: regress Amount_µl_R Age

----------------------------------------------------------------------------------

---------------------------------------------------------------------------------

.

. log close

name: <unnamed>

log: /Users/gyni/Extern/S Löhrer/Stata/Log/230706.smcl

log type: smcl

closed on: 6 Jul 2023, 10:24:27

----------------------------------------------------------------------------------
